# Genetic parameters for uniformity of harvest weight in Pacific white shrimp (*Litopenaeus vannamei*)

**DOI:** 10.1186/s12711-021-00621-6

**Published:** 2021-03-12

**Authors:** Silvia García-Ballesteros, Beatriz Villanueva, Jesús Fernández, Juan Pablo Gutiérrez, Isabel Cervantes

**Affiliations:** 1 Departamento de Mejora Genética Animal, INIA, 28040 Madrid, Spain; 2 Departamento de Producción Animal, Facultad de Veterinaria, Universidad Complutense de Madrid, 28040 Madrid, Spain

## Abstract

**Background:**

Uniformity of body weight is a trait of great economic importance in the production of white shrimp (*Litopenaeus vannamei*). A necessary condition to improve this trait through selective breeding is the existence of genetic variability for the environmental variance of body weight. Although several studies have reported such variability in other aquaculture species, to our knowledge, no estimates are available for shrimp. Our aim in this study was to estimate the genetic variance for weight uniformity in a farmed population of shrimp to determine the potential of including this trait in the selection program. We also estimated the genetic correlation of weight uniformity between two environments (selection nucleus and commercial population).

**Methods:**

The database contained phenotypic records for body weight on 51,346 individuals from the selection nucleus and 38,297 individuals from the commercial population. A double hierarchical generalized linear model was used to analyse weight uniformity in the two environments. Fixed effects included sex and year for the nucleus data and sex and year-pond combination for the commercial data. Environmental and additive genetic effects were included as random effects.

**Results:**

The estimated genetic variance for weight uniformity was greater than 0 (0.06 ± 0.01) in both the nucleus and commercial populations and the genetic coefficient of variation for the residual variance was 0.25 ± 0.01. The genetic correlation between weight and weight uniformity was close to zero in both environments. The estimate of the genetic correlation of weight uniformity between the two environments (selection nucleus and commercial population) was 0.64 ± 0.06.

**Conclusions:**

The existence of genetic variance for weight uniformity suggests that genetic improvement of this trait is possible. Selection for weight uniformity should not decrease weight, given the near zero genetic correlation between these two traits. The strong genetic correlation of weight uniformity between the two environments indicates that response to selection for uniformity in the nucleus will be at least partially transmitted to the commercial population if this trait is included in the breeding goal.

## Background

Shrimp production is an important economic activity in the aquaculture industry and ranks third in value, after salmon and trout. The white-leg shrimp (*Litopenaeus vannamei*) is the crustacean with the highest production level, i.e. thousands of tons of live weight worldwide [1]. In 2016, production of this species accounted for 53% of the total crustacean production and is continuously increasing. In fact, its production increased by 55% from 2010 to 2016 [1]. In addition, due to the growing demand, especially from developed countries, the price of farmed shrimp has increased in recent years.

Once production management conditions are controlled, the development of genetic improvement programs is one of the key factors for increased production efficiency and competitiveness. Currently, most of the selective breeding programs for shrimp focus on improving growth traits only [2, 3]. However, as growth rate increases and production intensifies [4, 5], other traits related to the quality and uniformity of the final product gain importance for both consumers and producers [6]. Shrimp are graded and classified according to standards that are defined in high-quality marketing evaluations, and are mainly determined by their physical characteristics and uniformity of size [7, 8]. In particular, shrimp are graded according to their size and count per unit of weight. Prices between size categories vary widely, and a larger number of shrimp per weight unit (i.e., of smaller size) results in a price reduction [9, 10]. Therefore, increasing the consistency of size within a specific count range can increase profit margins in the shrimp industry [10]. In addition, large variation in body size can cause competition among shrimp (dominance hierarchies), which negatively affects growth rate, mortality, and feed efficiency, and increases the need for management practices such as size grading [11]. Another indirect benefit of improving uniformity is its potential to improve resilience, which is defined as the ability of an animal to maintain performance in spite of environmental perturbations [11]. For all these reasons, and given that weight is genetically highly correlated with size, uniformity of weight is a clear candidate trait to be included in shrimp breeding programs.

Weight uniformity depends on the sensitivity of an individual to macro- and micro-environmental factors. Macro-environmental factors are measurable factors such as temperature, seasonality, diet and management, whereas micro-environmental factors are non-mensurable animal-specific factors within a given macro-environment. A necessary condition to increase weight uniformity, is the existence of genetic variance for response to such micro-environmental factors [12–14], such that individuals with genotypes that make them less sensitive to environmental disturbances will have more homogeneous offspring and show less environmental within-family variance. Several quantitative genetic models that account for genetic variance of the residual (environmental) variance have been developed [15–17], so-called heterogeneous residual variance models.

The existence of a genetic component for environmental variance has been demonstrated for different species of farmed mammals for traits such as weight and litter size [18–20]. However, in spite of the great interest to improve uniformity of aquaculture products, studies in aquaculture species are very scarce (see recent review by de Souza et al. [11]), and to our knowledge, there is very little information on weight uniformity in crustaceans [21].

In aquaculture selective breeding programs, the breeding nucleus (in which selection is performed) is usually kept separate from the commercial population that is composed of individuals destined for sale in the market. In aquaculture, the macro-environmental rearing conditions can differ greatly between the nucleus and the commercial population. Thus, if genotype-by-environment interactions exit, genetic improvement achieved in the nucleus may not be fully translated to the commercial population. In fact, in many aquaculture species, growth traits show a significant re-ranking of genotypes in different environments [22]. For body weight uniformity, Sae-Lim et al. [23] showed a moderate re-ranking of families in different environments in rainbow trout.

The aim of this study was to estimate the genetic variance of body weight uniformity in a farmed population of Pacific white shrimp (*Litopenaeus vannamei*), using a heterogeneous residual variance model. In addition, to investigate whether selecting for increased weight uniformity in the breeding nucleus leads to improvement of uniformity in the commercial population, the genetic correlation of weight uniformity between the two environments (selection nucleus and commercial population) was estimated.

## Methods

### Data

The data used in this study were obtained from the CAMANICA S.A. company, which is based in Nicaragua and is carrying out a breeding program in shrimp with discrete generations. Selection is for body weight and the number of selected individuals per generation is 300 (150 males and 150 females). Each male is mated (through artificial fertilization) with a single female, and each female is mated to a single male. Once shrimp reach the appropriate size, a random sample of individuals per family (between 150 and 200 depending on the year) are tagged, with half of them being individually tagged with eye-rings and assigned to the nucleus ($N$) and the other half being tagged at the family level with elastomers and assigned to the commercial population ($C$). Within the nucleus, all families are reared in the same tank. However, in the commercial population, three to four ponds that are located in different geographical zones are used per generation, with each family equally represented in each pond. Environmental conditions differ greatly between the nucleus and the commercial populations. Thus, weight in the selection nucleus and weight in the commercial population are considered as two different traits.

The data used here are from three consecutive generations and 425 families. The total number of individuals with phenotypic records for body weight at harvest was 89,643, of which 51,346 belonged to the nucleus and 38,297 belonged to the commercial population. Harvest time was established by estimating the days required to reach an average weight of 15 g in the nucleus. This time was set for both commercial (all ponds) and for the nucleus environments. However, for management reasons, recording the phenotypes of all shrimp can take a few days. Sex, year and pond were also recorded. A description of the number of shrimp in each category and environment is in Table 1. The average number of offspring with harvest body weight recorded per family was 205, with a standard deviation of 43.

**Table 1:** Description of the data structure in the nucleus ($N$) and in the commercial population ($C$)

	$N$	$C$	Total
Number of shrimp	51,346	38,297	89,643
Males	25,456	17,327	42,783
Females	25,890	20,970	46,860
N_l1_	16,144	2874; 3436; 3751	26,205
N_l2_	20,050	2870; 4949; 4738	32,607
N_l3_	15,152	3142; 3411; 2536; 6590	30,831
N_off_ (min–max)	118 (23–177)	88 (21–178)	205 (47–299)
A_wt_ (g)	28; 34; 33	19; 20; 23	–
SD_wt_ (g)	4.34; 5.65; 5.04	2.92; 3.03; 3.24	–

N_lx_: number of shrimp in level x of fixed effects (year in $N$ and year-pond in $C$); N_off_: average number of offspring per family; A_wt_: average weight per year; SD_wt_: standard deviation of weight per year

### Evaluation method

Genetic parameters for weight and weight uniformity (defined as the environmental variability of body weight) in each environment ($N$ and $C$) were estimated using double hierarchical generalized linear models (DHGLM) [24, 25]. As repeated observations are needed to deal with analysis of individual animal variances, the harvest weight variability was attributed to the family in this study. The family was identified by the mother [26–28] but it also contains a sire effect due to the single pair mating design, resulting in the sire and dam effects to be confounded. Thus, although harvest weight itself was considered as a trait of the individual, its genetic effect was modelled as the sum of the dam plus sire effects. This dam model has been shown to be highly successful to modify environmental variability of birth weight in a mice population under a single pair mating design, after 7 [27] and 17 [28] generations of selection. The analyses were performed separately for $N$ and $C$ using univariate models (including weight and its uniformity), and also jointly using a multivariate model. The same fixed and random effects were used for weight and its uniformity for both $N$ and $C$. Models were fitted using the ASReml software [29]. The equation for the multivariate DHGLM model was:

$$\left[ {\begin{array}{*{20}c} {\begin{array}{*{20}c} {{\mathbf{y}}_{N} } \\ {{\mathbf{d}}_{N} } \\ {{\mathbf{y}}_{C} } \\ \end{array} } \\ {{\mathbf{d}}_{C} } \\ \end{array} } \right] = \left[ {\begin{array}{*{20}c} {\begin{array}{*{20}c} {{\mathbf{X}}_{N} } \\ 0 \\ \end{array} } \\ {\begin{array}{*{20}c} 0 \\ 0 \\ \end{array} } \\ \end{array} \begin{array}{*{20}c} {\begin{array}{*{20}c} 0 \\ {{\mathbf{X}}_{N}^{*} } \\ \end{array} } \\ {\begin{array}{*{20}c} 0 \\ 0 \\ \end{array} } \\ \end{array} \begin{array}{*{20}c} {\begin{array}{*{20}c} 0 \\ 0 \\ \end{array} } \\ {\begin{array}{*{20}c} {{\mathbf{X}}_{C} } \\ 0 \\ \end{array} } \\ \end{array} \begin{array}{*{20}c} {\begin{array}{*{20}c} 0 \\ 0 \\ \end{array} } \\ {\begin{array}{*{20}c} 0 \\ {{\mathbf{X}}_{C}^{*} } \\ \end{array} } \\ \end{array} } \right]\left[ {\begin{array}{*{20}c} {\begin{array}{*{20}c} {{\mathbf{b}}_{N} } \\ {{\mathbf{b}}_{N}^{*} } \\ \end{array} } \\ {\begin{array}{*{20}c} {{\mathbf{b}}_{C} } \\ {{\mathbf{b}}_{C}^{*} } \\ \end{array} } \\ \end{array} } \right] + \left[ {\begin{array}{*{20}c} {\begin{array}{*{20}c} {{\mathbf{Z}}_{N} } \\ 0 \\ \end{array} } \\ {\begin{array}{*{20}c} 0 \\ 0 \\ \end{array} } \\ \end{array} \begin{array}{*{20}c} {\begin{array}{*{20}c} 0 \\ {{\mathbf{Z}}_{N}^{*} } \\ \end{array} } \\ {\begin{array}{*{20}c} 0 \\ 0 \\ \end{array} } \\ \end{array} \begin{array}{*{20}c} {\begin{array}{*{20}c} 0 \\ 0 \\ \end{array} } \\ {\begin{array}{*{20}c} {{\mathbf{Z}}_{C} } \\ 0 \\ \end{array} } \\ \end{array} \begin{array}{*{20}c} {\begin{array}{*{20}c} 0 \\ 0 \\ \end{array} } \\ {\begin{array}{*{20}c} 0 \\ {{\mathbf{Z}}_{C}^{*} } \\ \end{array} } \\ \end{array} } \right]\left[ {\begin{array}{*{20}c} {\begin{array}{*{20}c} {{\mathbf{u}}_{m,N} } \\ {{\mathbf{u}}_{m,N}^{*} } \\ \end{array} } \\ {\begin{array}{*{20}c} {{\mathbf{u}}_{m,C} } \\ {{\mathbf{u}}_{m,C}^{*} } \\ \end{array} } \\ \end{array} } \right] + \left[ {\begin{array}{*{20}c} {\begin{array}{*{20}c} {{\mathbf{e}}_{N} } \\ {{\mathbf{e}}_{N}^{*} } \\ \end{array} } \\ {\begin{array}{*{20}c} {{\mathbf{e}}_{C} } \\ {{\mathbf{e}}_{C}^{*} } \\ \end{array} } \\ \end{array} } \right],$$

where subscript $N$ refers to the selection nucleus and $C$ to the commercial population, $*$ indicates parameters that are associated with the residual variance (uniformity of body weight), ${\mathbf{y}}$ is the vector of body weight phenotypes, ${\mathbf{d}}$ is the vector of residual variances, with elements $d_{i} = \hat{e}_{i}^{2} /\left( {1 - h_{i} } \right)$, where $\hat{e}_{i}^{2}$ is the squared residual estimate of body weight in each environment ($N$ or $C$) and $h_{i}$ is the $i$th diagonal element in the hat matrix of ${\mathbf{y}}$ for that trait [24], ${\mathbf{b}}$ and ${\mathbf{b}}^{*}$ are vectors of fixed effects (sex and year for $N$ and sex and the year-pond interaction for $C$), ${\mathbf{X}}$ and ${\mathbf{X}}^{*}$ are incidence matrices associated with the fixed effects, ${\mathbf{u}}_{m}$ and ${\mathbf{u}}_{m}^{*}$ are vectors of additive genetic effects of the mother plus the father, and ${\mathbf{Z}}$ and ${\mathbf{Z}}^{*}$ are incidence matrices associated with the random genetic effects. The distribution of the additive genetic effects was as follows:

$$\left[ {\begin{array}{*{20}c} {\begin{array}{*{20}c} {{\mathbf{u}}_{m, N} } \\ {{\mathbf{u}}_{m,N}^{*} } \\ \end{array} } \\ {\begin{array}{*{20}c} {{\mathbf{u}}_{m,C} } \\ {{\mathbf{u}}_{m,C}^{*} } \\ \end{array} } \\ \end{array} } \right] \sim {\mathbf{N}}\left( {\begin{array}{*{20}c} 0 \\ 0 \\ 0 \\ 0 \\ \end{array} ,\;\left[ {\begin{array}{*{20}c} {\sigma_{{u_{m,N} }}^{2} } & {\rho \sigma_{{u_{m,N} }} \sigma_{{u_{m,N}^{*} }} } & {\rho \sigma_{{u_{m,N} }} \sigma_{{u_{m,C} }} } & {\rho \sigma_{{u_{m,N} }} \sigma_{{u_{m,C}^{*} }} } \\ {} & {\sigma_{{u_{m,N}^{*} }}^{2} } & {\rho \sigma_{{u_{m,N}^{*} }} \sigma_{{u_{m,C} }} } & {\rho \sigma_{{u_{m,N}^{*} }} \sigma_{{u_{m,C}^{*} }} } \\ {sym} & {} & {\sigma_{{u_{m,C} }}^{2} } & {\rho \sigma_{{u_{m,C} }} \sigma_{{u_{m,C}^{*} }} } \\ {} & {} & {} & {\sigma_{{u_{m,C}^{*} }}^{2} } \\ \end{array} } \right] \otimes {\mathbf{A}}} \right),$$

where ${\mathbf{A}}$ is the numerator relationship matrix based on pedigree information available for the mothers (three generations), $\sigma_{{u_{m} }}^{2}$ and $\sigma_{{u_{m}^{*} }}^{2}$ are the additive genetic variances for body weight and its uniformity, respectively, $\rho$ is the genetic correlation coefficient and $\otimes$ represents the Kronecker product. Residuals of ${\mathbf{y}} \left( {\mathbf{e}} \right)$ and ${\mathbf{d}} \left( {{\mathbf{e}}^{*} } \right)$ were assumed to be independent and normally distributed:

$$\left[ {\begin{array}{*{20}c} {\begin{array}{*{20}c} {{\mathbf{e}}_{ N} } \\ {{\mathbf{e}}_{N}^{*} } \\ \end{array} } \\ {\begin{array}{*{20}c} {{\mathbf{e}}_{C} } \\ {{\mathbf{e}}_{C}^{*} } \\ \end{array} } \\ \end{array} } \right] \sim {\mathbf{N}}\left( {\begin{array}{*{20}c} {\begin{array}{*{20}c} 0 \\ 0 \\ \end{array} } \\ {\begin{array}{*{20}c} 0 \\ 0 \\ \end{array} } \\ \end{array} , \left[ {\begin{array}{*{20}c} {\begin{array}{*{20}c} {{\mathbf{W}}_{N}^{ - 1} \sigma_{e}^{2} } \\ 0 \\ \end{array} } \\ {\begin{array}{*{20}c} 0 \\ 0 \\ \end{array} } \\ \end{array} \begin{array}{*{20}c} {\begin{array}{*{20}c} 0 \\ {{\mathbf{W}}_{N}^{* - 1} \sigma_{{e^{*} }}^{2} } \\ \end{array} } \\ {\begin{array}{*{20}c} 0 \\ 0 \\ \end{array} } \\ \end{array} \begin{array}{*{20}c} {\begin{array}{*{20}c} 0 \\ 0 \\ \end{array} } \\ {\begin{array}{*{20}c} {{\mathbf{W}}_{C}^{ - 1} \sigma_{e}^{2} } \\ 0 \\ \end{array} } \\ \end{array} \begin{array}{*{20}c} {\begin{array}{*{20}c} 0 \\ 0 \\ \end{array} } \\ {\begin{array}{*{20}c} 0 \\ {{\mathbf{W}}_{C}^{* - 1} \sigma_{{e^{*} }}^{2} } \\ \end{array} } \\ \end{array} } \right]} \right),$$

where ${\mathbf{W}} = {\text{diag}}(\hat{d}^{ - 1}$), ${\mathbf{W}}^{*} = {\text{diag}}\left( {\frac{{1 - h_{i} }}{2}} \right)$, and $\sigma_{e}^{2}$ and $\sigma_{{e^{*} }}^{2}$ are scaling variances (set equal to 1). The multivariate DHGLM used a weighted gamma GLM fitted with response ${\mathbf{d}}$ and weights ${\mathbf{W}}^{*}$ and ${\mathbf{W}}$ [24]. For the univariate DHGLM, only the parameters for one environment ($N$ or $C$) were included in the model.

### Parameters evaluated

The estimated parameters included the genetic variance for both weight and its uniformity in $N$ and $C$, heritabilities, genetic correlations between traits (see below), and the genetic coefficient of variation for the residual variance ($GCV_{e}$) of weight uniformity. The latter was computed using the approximation $\sqrt {\sigma_{{u_{m}^{*} }}^{2} }$ because $\sigma_{{u_{m}^{*} }}^{2}$ is on an exponential scale [17, 20].

Genetic correlations were estimated (i) for weight between the two environments ($N$ and $C$); (ii) for weight uniformity between the two environments; (iii) between weight and weight uniformity within each environment ($N$ or $C$); and (iv) between weight in one environment and weight uniformity in the other environment.

Heritability of weight uniformity ($h_{v}^{2}$) was estimated for $N$ and $C$ as the proportion of phenotypic variance in body weight that was due to the genetic variance of the residual variance [17, 20]: $h_{v}^{2} = \sigma_{{u^{*} }}^{2} /\left( {2\sigma_{P}^{4} + 3\sigma_{{u^{*} }}^{2} } \right)$, where $\sigma_{P}^{2} = 2\sigma_{u}^{2} + \sigma_{e}^{2}$. Because $\sigma_{{u^{*} }}^{2}$ is on an exponential scale, it was converted to an additive scale following Mulder et al. [17], i.e., $\sigma_{{u_{a}^{*} }}^{2} = \sigma_{e}^{4} {\text{exp}}\left( {2\sigma_{{u^{*} }}^{2} } \right) - \sigma_{{e_{a} }}^{4}$, where $\sigma_{{e_{a} }}^{4}$ equals $\sigma_{e}^{2} {\text{exp}}\left( {\frac{1}{2}\sigma_{{u^{*} }}^{2} } \right)$.

Heritability of weight was estimated for $N$ and $C$. The estimated genetic variance $(\sigma_{{u_{m} }}^{2} )$ refers to sire plus dam effects and equals to half of the total genetic variance $\left( {\sigma_{u}^{2} } \right)$. Under heteroscedastic models, heritability (i.e., the usual ratio of additive to phenotypic variance) of the mean (weight) is not unique because it depends on the value of the residual variance, which in turn is conditioned to the levels of the environmental effects (${\varvec{b}}^{*}$) [19, 26, 30]:

$$h_{i}^{2} = \frac{{\sigma_{u}^{2} }}{{\sigma_{{u_{m} }}^{2} + e^{{\left( {{\text{Xb}}_{i}^{*} + {\raise0.7ex\hbox{$1$} \!\mathord{\left/ {\vphantom {1 2}}\right.\kern-\nulldelimiterspace} \!\lower0.7ex\hbox{$2$}}\sigma_{{u_{m}^{*} }}^{2} } \right)}} }}.$$

A global heritability was estimated for weight in $N$ and $C$ using a global residual variance obtained by averaging across fixed effect solutions. Estimates of heritability for each combination of level $l$ of a systematic effect $s$ were also obtained, using:

$$\sigma_{{e_{sl} }}^{2} = e^{{\mathop \sum \limits_{i = 1,systematics}^{i \ne s} \left( {\mathop \sum \limits_{{j = 1,n_{s} }} \frac{{\hat{b}_{ij} }}{{n_{s} }}} \right) + \hat{b}_{sl} + {\raise0.7ex\hbox{$1$} \!\mathord{\left/ {\vphantom {1 2}}\right.\kern-\nulldelimiterspace} \!\lower0.7ex\hbox{$2$}}\sigma_{{u_{m}^{*} }}^{2} }} ,$$

resulting heritability equal to:

$$h_{sl}^{2} = \frac{{\sigma_{u}^{2} }}{{\sigma_{{u_{m} }}^{2} + \sigma_{{e_{sl} }}^{2} }}.$$

These estimates of heritability were obtained using a Bayesian approach with the GSEVM software [31] and using univariate models with the same systematic and random effects as included when using a frequentist approach. Results from the Bayesian approach were obtained by averaging the results from a Markov chain of Monte Carlo (MCMC) chain of 1,000,000 iterations, sampling one of each 100 iterations, following a burn-in of 100,000. Inferences were based on probabilities obtained from the marginal posterior distributions of the parameters or combinations of parameters and the estimates are the means of these marginal posterior distributions.

## Results

### Genetic variance components and heritabilities

Estimates of variance components and genetic parameters for weight and its uniformity in the two environments ($N$ and $C$) are in Table 2 for the univariate and multivariate models, which led to very similar results. The estimate of the additive genetic variance for body weight was substantially larger in $N$ than in $C$ but the estimated heritabilities were similar in the two environments. The estimate of the additive genetic variance for weight uniformity was different from 0 for both $N$ and $C$ (0.06 ± 0.01) and the estimate of heritability was 0.02 for both. Thus, the genetic coefficient of residual variation (weight uniformity) was the same in the two environments (around 0.25), as was the estimate of the global heritability of body weight (around 0.39).

**Table 2:** Estimates (standard errors) of variance components and genetic parameters for weight and weight uniformity in different environments (nucleus, $N$, and commercial, $C$) obtained from multivariate and univariate DHGLM models

	Multivariate		Univariate	
	$N$	$C$	$N$	$C$
Weight				
$\sigma_{{u_{m} }}^{2}$	4.45 (0.33)	1.60 (0.12)	4.42 (0.33)	1.60 (0.12)
$\sigma_{u}^{2}$	8.90	3.20	8.85	3.19
$\sigma_{e}^{2}$	19.06	6.64	18.98	6.60
$\sigma_{P}^{2}$	23.51	8.23	23.40	8.19
$h^{2}$	0.38	0.39	0.38	0.39
Weight uniformity				
$\sigma_{{u_{m}^{*} }}^{2}$	0.06 (0.01)	0.06 (0.01)	0.06 (0.01)	0.06 (0.01)
$h_{v}^{2}$	0.02	0.02	0.02	0.02
$GCV_{e}$	0.25 (0.01)	0.24 (0.01)	0.25 (0.01)	0.23 (0.02)

DHGLM: double hierarchical generalized linear model. $\sigma_{{u_{m} }}^{2} , \sigma_{{u_{m}^{*} }}^{2}$: additive variances of the mother plus father for body weight and its uniformity, respectively; $\sigma_{u}^{2}$: additive variance for body weight defined as 2*$\sigma_{{u_{m} }}^{2}$; $\sigma_{e}^{2}$: residual variance; $\sigma_{P}^{2} :$ phenotypic variance; $h^{2}$, $h_{v}^{2}$: heritabilities of weight and its uniformity, respectively; $GCV_{e}$: genetic coefficient of residual variation for weight uniformity

**Fig. 1:**
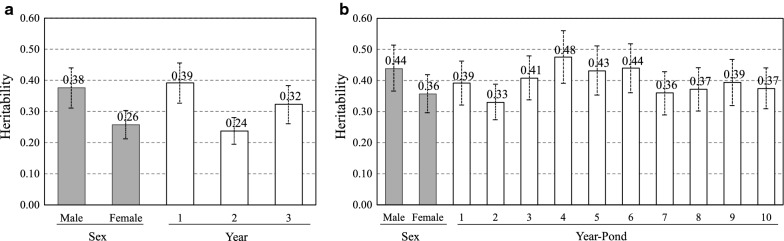
Estimates of heritability (mean and a posteriori standard deviation) of harvest weight for each level of residual systematic effects. Systematic effects were sex and year for the nucleus (**a**) and sex and year-pond for the commercial population (**b**)

Figure 1 shows the estimates of heritabilities (mean and standard deviation of the marginal posterior distributions obtained using the Bayesian approach) for the different levels of systematic effects. These included sex (male or female) and year (from 1 to 3) for weight in $N$ and sex and year-pond (from 1 to 10) for weight in $C$. In $N$, the estimate of heritability for males was about 46% higher than for females (0.38 vs. 0.26). In $N$, the estimate of heritability was highest for the first year and lowest for the second year. The probabilities (obtained from the marginal posterior distributions) that heritability was lower in the third year than in the first year heritability and higher than in the second year were 99.8 and 100%, respectively. Differences in estimates of heritability within levels of systematic effects were also observed for $C$. In $C$, the probability that the heritability was higher for males than for females was 99.9%, in agreement with the results obtained for $N$. For the year-pond effect in $C$, level 2 shows the lowest estimate of heritability with a probability of 85 to 100% of being lower than for the other year-pond levels.

### Genetic correlations

Estimates of the genetic correlation between weight and its uniformity within and between the $N$ and $C$ environments were also very similar for the univariate and multivariate models (Table 3). In $N$, the estimate of the genetic correlation between weight and its uniformity was positive and relatively low (0.11) but higher than in $C$, for which the estimate was not significantly different from 0.

**Table 3:** Estimates (standard errors) of genetic correlations between weight and weight uniformity within and between environments (nucleus, $N$, and commercial, $C$) obtained from multivariate (above the diagonal) and univariate (below the diagonal) DHGLM models

	$N$		$C$	
	Weight	Uniformity	Weight	Uniformity
$$N$$				
Weight		0.11 (0.06)	0.61 (0.03)	0.06 (0.07)
Uniformity	0.11 (0.06)		0.01 (0.06)	0.64 (0.06)
$$C$$				
Weight	**–**	**–**		0.05 (0.07)
Uniformity	**–**	**–**	0.07 (0.07)	

DHGLM: double hierarchical generalized linear model

Estimates of the genetic correlation between weight in $N$ and weight in $C$ and between weight uniformity in $N$ and weight uniformity in $C$ were both positive (about 0.60). Estimates of the genetic correlation between weight in $N$ and weight uniformity in $C$ and between weight in $C$ and weight uniformity in $N$ were not significantly different from 0.

### Ranking of families for weight uniformity in the two environments

Spearman correlations between estimated breeding values for weight uniformity in the nucleus and the commercial population were obtained by assuming a multivariate heterogeneous model. The correlations obtained using all (100%) candidate families (i.e., in $N$), or families in the top 50, top 20, and top 10% were 0.87, 0.79, 0.77, and 0.76, respectively. ‘Top’ families in this case were those with the highest estimated breeding value for uniformity (lowest weight variability). As expected, correlations were lower when the number of top families decreased, which indicates that a higher re-ranking between environments $N$ and $C$ for weight uniformity is expected in the best families. Regardless of this, correlations were in general relatively high.

## Discussion

### Genetic variances and heritabilities

Although weight uniformity is a very relevant trait with the potential of being included in shrimp breeding programs, there is very little information on the existence of genetic variation for this trait [21]. To our knowledge, this is the first study that uses a double hierarchical generalized linear model to estimate genetic variance for body weight uniformity in shrimp, and constitutes a first step to investigate the possibility of including this trait in the breeding goal. This is important since the weight uniformity evaluated here was individual sensitivity to micro-environmental disturbances. Estimates of the additive genetic variance, heritability, and genetic coefficient of residual variation for weight uniformity that were obtained for this Pacific white shrimp population in the nucleus, in which selection takes place, were all different from 0, which indicates that genetic improvement for this trait is possible. In addition, the genetic correlation of weight uniformity between the nucleus and the commercial population was relatively high, which indicates that improvement obtained in the nucleus would be partially transmitted to the commercial population, with the economic benefits that this would entail.

Estimates of the global heritability for body weight at harvest in $N$ and $C$ (0.38 and 0.39) were within the range of those found in the literature for shrimp [4, 5, 21]. More important, is the fact that estimates of the additive genetic variance for uniformity of weight and for the residual heritability were also in the range of those described for shrimp [21], other aquaculture [23, 32–35], and terrestrial [11] species. The estimate of the genetic coefficient of residual variation was lower than that reported for Nile tilapia [33] but similar to that reported for trout [23], and within the range of that reported for terrestrial animals [11]. This indicates the existence of genetic variation in micro-environmental sensitivity among full sib families (dam plus sire effects), which implies that the phenotypes of offspring of different families will be differentially affected by the environment. Thus, our results show that the potential of genetic selection to improve weight uniformity is similar to that for other species. Selection for improving homogeneity of birth weight in rabbits [36] or environmental variance for birth weight in mice [27, 28] has been shown to be successful, with estimates of genetic parameters similar to those obtained in this study.

Response to selection on a trait depends on the heritability of the trait, among other factors. Under a heteroscedastic model, estimates of heritability can be obtained for each level of the systematic effects [19, 30]. Our results show that estimates of heritability differed between levels of sex and year (or year-pond). Formoso-Rafferty et al. [26] suggested to restrict the information (phenotypes) to the levels of the systematic effects that have the higher heritabilities in order to decrease the residual variance. However, they also recognized that ignoring information from levels that are associated with higher residual variance could have a negative impact on the accuracy of estimated breeding values and thus, on response to selection. In practice, funds to record phenotypes are limited and it might be better to focus on recording phenotypes for traits with the highest heritability.

### Genetic correlations

In order to evaluate the potential economic benefit of including weight uniformity in the breeding goal, correlations with other traits that are currently in the breeding goal, such as body weight, must be estimated. The ideal scenario would be the existence of a negative genetic correlation between weight and its variability because it would facilitate selection for higher weight and more uniformity. In aquaculture, estimates of the genetic correlation between weight and its variability vary largely in the literature, i.e. between − 0.16 and 0.79 [23, 32–35]. Our estimate was not significantly different from 0 (Table 3), which indicates that it may not be difficult to improve weight and weight uniformity simultaneously through a selection index. This would require the economic value for uniformity to be determined, which is unknown at this point.

It is very important that genetic improvements made in the nucleus are transferred to the commercial population that is composed of individuals for sale in the market. Thus, a high genetic correlation between the nucleus and commercial environments for traits that are selected for in the nucleus is desirable. This is not always the case because, although conditions are intended to be similar in the two environments, this is not usually feasible. In particular, in aquaculture species, some environmental factors are more important than others in affecting the re-ranking of individuals based on their estimated breeding values. In their review on genotype-by-environment interactions, Sae-Lim et al. [22] found that differences in temperature, rearing system, or stocking density had a greater influence on the re-ranking of individuals for growth traits (estimates of the genetic correlation between environments ranged from 0.36 from 0.56) than differences in farm location (estimates of the genetic correlation ranged from 0.58 to 0.66). In our study, estimates of the genetic correlation between environments $N$ and $C$ were 0.61 for weight and 0.64 for weight uniformity, which are within the range reported for weight in other aquaculture species [22]. Our estimate of the genetic correlation of weight between environments was lower than that reported for shrimp by Castillo-Juarez et al. [5] but within the range of that reported by Gitterle et al. [4]. Our study provides, for the first time, an estimate of the genetic correlation of weight uniformity between different environments for shrimp, and it is similar to that reported for trout [23].

### Statistical models

Estimates and their standard errors of variance components and genetic parameters for weight uniformity and for the genetic correlation of weight with its uniformity obtained using univariate versus multivariate models were very similar (Table 2). In fact, Pearson’s correlations of estimated breeding values for weight uniformity between the univariate and multivariate models were high (0.87 in $N$ and 0.80 $C$).

To estimate the genetic variation of the residual variance, repeated observations are needed. However, traits such as body weight at harvest are recorded only once. Repeated measurements can be obtained within families by assigning the trait to the mother or the family rather than to the individual. Some studies that fitted the sire and dam effects separately for both the mean (weight) and its uniformity, obtained higher accuracies and less bias for the estimated variance components than when the individual genetic effect was fitted for the mean and the sire and dam effects were fitted for uniformity [23, 32]. In our study, convergence was not obtained when this approach was used because single pair matings were performed and the sire genetic effect could not be distinguished from the dam genetic effect. Thus, to estimate the genetic variance of the environmental variability, the mean and its uniformity were attributed to the mother [26–28]. However, the mother identification refers also to the father given that they are confounded. Maternal and common environmental effects were not included for the mean in the model because of the data structure, which originates from single pair mating with no replication of tanks per family. The maternal effect is considered to be the part of the environmental effect that affects performance of the offspring that depends on the environment provided by their mother (composition of the eggs among others) [37]. Maternal effects are significant for harvest weight in shrimp [4, 5] and other aquaculture species [34, 38, 39] but of low magnitude. The common environmental family effect originates from rearing families in different tanks during the early stages of growth (until individuals reach a sufficient size for tagging), and thus is common to members of the same family [40]. Many studies have shown that the proportion of phenotypic variance due to common environmental effects, although significantly different from 0 in shrimp [41] and other aquaculture species [35, 40, 42, 43], is of low magnitude. Some studies [42, 44] have suggested that common environmental effects are difficult to separate from family genetic effects, even with data from nested designs. In order to include these random effects in the model and avoid confounding and convergence problems, a possible solution would be to replicate tanks per family or perform factorial matings. The few studies that have included common environmental effects for weight uniformity either did not achieve convergence or did not obtain estimates significantly higher than 0 [32, 33, 35]. Thus, even if maternal and common environmental effects exist, they would probably explain only a small proportion of the phenotypic variance.

## Conclusions

Our results show that genetic variability for the environmental variance of weight at harvest exists in shrimp, both in the selection nucleus and in the commercial population. The coefficients of genetic residual variation for these traits (uniformity measured in the nucleus and in the commercial population) were large enough to conclude that response to selection could be obtained if these traits were included in the breeding program. Including weight uniformity should not decrease weight since the genetic correlation between the two traits was not significantly different from zero. Further investigation is necessary to determine what is the best combination of these traits in the selection index to reach the greatest economic benefit. Based on the genetic correlation of weight uniformity between the two environments estimated here, selection in the nucleus will be transmitted to the commercial population.

## Data Availability

The data that support the findings of this study are available from the CAMANICA S.A. company but restrictions apply to the availability of these data, which were used under license for the current study, and thus are not publicly available. However, data are available from the authors upon reasonable request and with permission of CAMANICA S.A.
